# Real world effectiveness of tixagevimab/cilgavimab (Evusheld) in the Omicron era

**DOI:** 10.1371/journal.pone.0275356

**Published:** 2023-04-27

**Authors:** Benjamin Chen, Nina Haste, Nancy Binkin, Nancy Law, Lucy E. Horton, Nancy Yam, Victor Chen, Shira Abeles

**Affiliations:** 1 Department of Medicine, Infectious Diseases and Global Public Health, University of California San Diego, San Diego, California, United States of America; 2 Department of Pharmacy, University of California San Diego, La Jolla, California, United States of America; 3 The Herbert Wertheim School of Public Health and Human Longevity, University of California San Diego, La Jolla, California, United States of America; Consejo Nacional de Investigaciones Cientificas y Tecnicas, ARGENTINA

## Abstract

**Background:**

Pre-exposure prophylaxis for COVID-19 with tixagevimab/cilgavimab (T/C) received Emergency Use Authorization (EUA) based on results of a clinical trial conducted prior to the emergence of the Omicron variant. The clinical effectiveness of T/C has not been well described in the Omicron era. We examined the incidence of symptomatic illness and hospitalizations among T/C recipients when Omicron accounted for virtually all local cases.

**Methods:**

Through retrospective electronic medical record chart review, we identified patients who received T/C between January 1 –July 31, 2022 within our quaternary referral health system. We determined the incidence of symptomatic COVID-19 infections and hospitalizations due to or presumed to be caused by early Omicron variants before and after receiving T/C (pre-T/C and post-T/C). Chi square and Mann-Whitney Wilcoxon two-sample tests were used to examine differences between the characteristics of those who got COVID-19 before or after T/C prophylaxis, and rate ratios (RR) and 95% confidence intervals (CI) were calculated to assess differences in hospitalization rates for the two groups.

**Results:**

Of 1295 T/C recipients, 105 (8.1%) developed symptomatic COVID-19 infection before receiving T/C, and 102 (7.9%) developed symptomatic disease after receiving it. Of the 105 patients who developed symptomatic infection pre-T/C, 26 (24.8%) were hospitalized, compared with six of the 102 patients (5.9%) who were diagnosed with COVID-19 post-T/C (RR = 0.24; 95% CI = 0.10–0.55; p = 0.0002). Seven of the 105 (6.7%) patients infected pre-T/C, but none of the 102 infected post-T/C required ICU care. No COVID-related deaths occurred in either group. The majority of COVID-19 cases among those infected pre-T/C treatment occurred during the Omicron BA.1 surge, while the majority of post-T/C cases occurred when Omicron BA.5 was predominant. In both groups, having at least one dose of vaccine strongly protected against hospitalization (pre-T/C group RR = 0.31, 95% CI = 0.17–0.57, p = 0.02; post-T/C group RR = 0.15; 95% CI = 0.03–0.94; p = 0.04).

**Conclusion:**

We identified COVID-19 infections after T/C prophylaxis. Among patients who received T/C at our institution, COVID-19 Omicron cases occurring after T/C were one-fourth as likely to require hospitalization compared to those with Omicron prior to T/C. However, due to the presence of changing vaccine coverage, multiple therapies, and changing variants, the effectiveness of T/C in the Omicron era remains difficult to assess.

## Introduction

COVID-19 vaccines have dramatically reduced the severity of disease caused by SARS-CoV-2 infection [1]. Immunocompromised patients, however, remain at risk for SARS-CoV-2 infections and, if infected, an increased risk of severe illness, hospitalization, and death. Although the overall mortality in immunocompromised patients with the SARS-CoV-2 Omicron variants has been shown thus far to be lower than with prior variants, studies have shown that hospitalization and prolonged duration of symptoms have remained high for this vulnerable population [2].

Tixagevimab/cilgavimab (AZD7442; Evusheld, subsequently referred to as T/C), a combination of two long-acting monoclonal antibodies, AZD8895 and AZD1061 (AstraZeneca), was shown in the PROVENT trial to reduce the risk of symptomatic COVID-19 infection by 83% compared with placebo among unvaccinated and relatively healthy patients [3]. In early December 2021, the Food and Drug Administration (FDA) granted an Emergency Use Authorization (EUA) of T/C for pre-exposure prophylaxis in moderately to severely immunocompromised adults and adolescents, and for those not eligible for COVID-19 vaccination due to a history of a severe reaction.

The authorization of T/C coincided with the emergence of the first Omicron variant, which resulted in previously effective monoclonal antibodies, including T/C, losing effectiveness [4–6]. Based on its reduced effectiveness against Omicron, the FDA recommended doubling the dose of T/C in February 2022 [7]. There is limited data available on the real-world effectiveness of T/C, especially in the Omicron era. T/C requires significant resources for administration due to its EUA status and associated institutional requirements for its administration. We therefore investigated COVID-19 infections and clinical outcomes in a cohort of immunocompromised patients who received T/C for pre-exposure prophylaxis within a single health system during a period when Omicron was the predominant circulating variant.

## Methods

The study was conducted at the University of California San Diego’s Health System (UC San Diego Health), a quaternary referral center serving a large population with many patients requiring complex subspecialty care. This system began administering T/C to patients in January 2022 according to the patient prioritization scheme set forth by the National Institute of Health’s COVID-19 Treatment Guidelines Panel [8]. Patients initially received 300 mg of drug, but the FDA recommended an increased dose for a total of 600 mg on February 24, 2022, based on *in vitro* studies using the Omicron variant [4–7, 9]. Patients who had received the 300 mg dose were advised to receive an additional “catch-up” dose.

### Study population and case definition

The study population consisted of any person age 18 years and above who received T/C at our institution between January 1, 2022 and July 31, 2022. To identify possible cases for inclusion in the study, we utilized the electronic medical records (EMR). Cohort members were considered to have had COVID-19 if they had a positive SARS CoV-2 test or if they had been prescribed one of the COVID-19 therapeutics (remdesivir (Veklury, Gilead), nirmatrelvir-ritonavir (Paxlovid, Pfizer), and neutralizing monoclonal antibodies (including REGEN-COV [casirivimab/imdevimab], (Regeneron); sotrovimab, (GlaxoSmithKline); and bebtelovimab, (Eli Lilly). For those patients with multiple positive tests, we performed a chart review to assess whether the repeat positive test was considered by clinical teams to have been consistent with a new infection.

Although we identified all cases in our study population between October 1, 2021 and July 31, 2022, the current analysis focuses on cases who were confirmed on the basis of genotyping to have an Omicron variant or who were highly likely, based on local epidemiologic trends, to have been infected with an Omicron variant. Based on San Diego Wastewater Surveillance data (SEARCH GitHub repository (https://searchcovid.info/dashboards/wastewater-surveillance/), Omicron first appeared in San Diego the last week of November and by the 19^th^ of December, 2021, it accounted for two thirds (67%) of all isolates in the wastewater. The 19^th^ of December was also the date of the first positive Omicron genotype in a member of the study population, after which only Omicron was identified. For that reason, we limited our analysis to cases occurring between December 19 2021 and July 31, 2022 when the study ended. At the end date, Omicron variants accounted for 100% of the wastewater viral load. We used EMR data to obtain each patient’s demographic and medical characteristics including history relevant to T/C eligibility, COVID immunization history, clinical course of COVID-19 infection during the study period including whether infection resulted in hospitalization, intensive care unit stay, or death, dates and doses of T/C receipt, date of first positive COVID-19 test, available COVID-19 genotype sequencing and COVID-specific antiviral and monoclonal antibody therapeutics received.

### Comparison of COVID-19 outcomes in patients infected before (“pre”) and after (“post”) having received T/C

To create a comparison group to better assess the effectiveness of T/C, we examined the characteristics and clinical outcomes of COVID-19 cases in this cohort for those who developed COVID-19 before receiving T/C (“pre-T/C”) and after receiving T/C (“post-T/C”). Numbers of cases, hospitalizations, and ICU admissions were compared between the pre-T/C and post-T/C groups. For the post-T/C group, we calculated the interval in days between last dose of T/C and date of first positive test or prescription of COVID-19 therapeutic. We used chi square and the Mann-Whitney Wilcoxon two-sample tests to examine differences between those who developed COVID-19 infection before T/C and after T/C prophylaxis.

### Evaluating SARS-CoV-2 cases within the epidemiological context of regional and temporal Omicron surges

We plotted cases and hospitalizations by week of diagnosis and T/C status to evaluate the temporal distribution of COVID-19 Omicron infections in relationship to time of T/C administration. To place the cases in the context of the COVID-19 patterns in the community and which variants were circulating in the San Diego area during the same time interval, we used data from the SEARCH GitHub repository to develop a graph of mean weekly wastewater concentrations of SARS-CoV-2 by variant type.

We examined the impact of T/C on rates of COVID-19 hospitalization and ICU admission by comparing the rates of each outcome among patients who received T/C before developing COVID-19 and those who developed it after receiving T/C. We calculated rate ratios, 95% confidence intervals, and two-tail p-values. For this analysis, we also examined the rates by underlying condition (hematologic malignancy, bone marrow transplantation, solid organ transplantation, and other, which included patients with underlying immunosuppression such as rheumatologic conditions or AIDS). We also calculated the E-values for all associations of interest in order to quantify the strength of an unmeasured confounder to explain away the observed association. The e-value quantifies the strength of an unmeasured confounder to explain away the observed association [10, 11]. Finally, we compared characteristics of patients hospitalized for COVID-19 in the pre-T/C and post-T/C groups as well as their hospitalization courses using two-tailed Fisher exact and the Mann-Whitney Wilcoxon two-sample test.

This study was determined by the UC San Diego Institutional Review Board to be exempt from Institutional Review Board requirements under categories 45 CFR 46.104(d), category 4.

## Results

### General patient characteristics

From January 1, 2022, through July 31, 2022, UC San Diego Health administered pre-exposure T/C prophylaxis to 1295 patients, of whom 9.2% received it in January (the majority of whom received “catch-up” doses in March), 18.8% in February, 17.1% in March, 23.3% in April, 14.0% in May, and the remaining 17.6% between June and July 2022.

The median age of the patients who received T/C was 59 years (range 18–99 years); 57.5% were male, 42.4% were female, and 0.1% were non-binary. The majority of T/C recipients were bone marrow transplant recipients or hematologic malignancy patients (47.7%). A total of 37.2% were solid organ transplant recipients, and 15.1% had other qualifying conditions.

### Characteristics of patients who developed COVID-19

Of the 1295 T/C recipients, 206 (15.9%) met the case definition of infection with the Omicron variant of COVID-19 during the study period. One patient developed COVID-19 infections twice, once before and once after receiving T/C, bringing the total cases to 207. Of the 207 infections, 105 (50.7%) developed COVID-19 before receiving T/C; the remaining 102 (49.3%) developed it after receiving T/C (Table 1). A total of 148/207 cases (71.5%) were identified based on positive test results, and the remaining 59/207 (28.5%) on the basis of having been prescribed a COVID-19 therapy.

**Table 1:** Characteristics of recipients of tixagevimab-cilgavimab (T/C) who developed COVID-19 infection between December 19, 2021 and July 31, 2022 when Omicron was the dominant circulating variant. Characteristics of T/C recipients who were infected prior to receiving T/C (pre-T/C) are compared with characteristics of T/C recipients who were diagnosed with COVID after having received any dose of T/C (post-T/C).

Patient Characteristic	Patients with COVID-19 infection pre-T/C (*n = 105*)	Patients with COVID-19 infection post-T/C (*n = 102*)	*p*-value
**Median age [range], years**	54.0 [18–79]	60.5 [25–99]	*p* = 0.008
**Patient gender**			N.S.
Male (%)	58 (55.2)	58 (56.9)	
Female (%)	47 (44.8)	44 (43.1)	
**Underlying conditions**			N.S.
Solid organ transplantation (%)	43 (41.0)	35 (34.3)	
Hematologic malignancy or bone marrow transplantation (%)	46 (43.8)	56 (54.9)	
Other (%)^•^	16 (15.2)	11 (10.8)	
**Number (%) of vaccine doses received prior to COVID diagnosis** ^**†**^			*p* < 0.001
0	15 (14.3)	3 (2.9)	
1–2	29 (27.6)	25 (24.8)	
3	53 (50.5)	36 (35.6)	
4	8 (7.6)	37 (36.6)	
**Therapeutics received (%)** *			*p* ≤ 0.001
Nirmatrelvir-Ritonavir	5 (4.8)	40 (39.2)	
Bebtelovimab	0 (0.0)	34 (33.3)	
Remdesivir	18* (17.1)	17 (16.7)	
Sotrovimab	57* (54.3)	2 (2.0)	
None	26 (24.8)	9 (8.8)	
**Number of patients hospitalized (%)**	26 (24.8)	6 (5.9)	*p* < 0.001
**Number of ICU admissions (%)**	7 (6.7)	0 (0.0)	p = 0.008
**Number of deaths from COVID-19 (%)**	0 (0.0)	0 (0.0)	—

^•^Including autoimmune conditions, HIV/AIDS, and solid tumor malignancy

^†^One patient’s vaccine history was not documented within the electronic medical record

*One patient was treated with both sotrovimab and remdesivir

**Abbreviations**:

ICU = Intensive Care Unit

**Fig 1:**
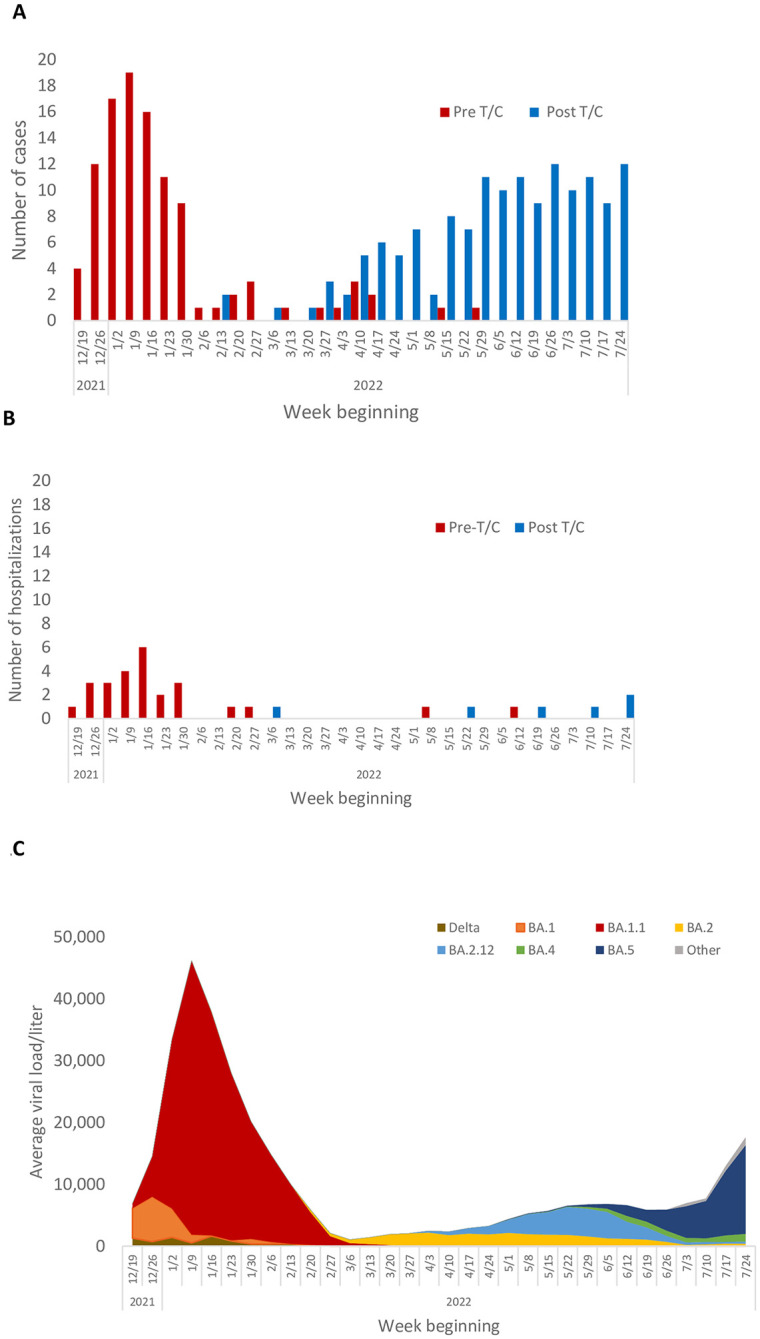
A. Cases of COVID-19 by week and T/C status at the time of COVID-19 diagnosis, UC San Diego Health, December 2021—July 2022. B. Hospitalizations for COVID-19 by week and T/C status at the time of COVID-19 diagnosis, UC San Diego Health, December 2021—July 2022. C. Average weekly SARS CoV-2 wastewater viral loads/liter by variant, San Diego County, December 2021 –July 2022.

The distribution of cases over time in our study cohort is shown in Fig 1A. It shows a sharp increase in cases in late December coinciding with the rapid emergence of the Omicron variant in the San Diego region (Fig 1C). The number of cases peaked in the first week of January, falling to a lower baseline level by the end of the month. All of the cases before and during the winter peak were among patients who had not yet received T/C. Fig 1B demonstrates a parallel rise in hospitalization during the winter peak, with all cases occurring among those who had not yet received T/C. The local circulating strain was initially Omicron BA.1, which was rapidly replaced by BA.1.1 (Fig 1C). In late April 2022, cases began to rise again, with wastewater data showing increasing levels of Omicron BA2.12 and subsequently BA.5.

We compared the 105 patients who developed COVID-19 due to Omicron pre-T/C with the 102 patients who developed it post T/C (Table 1). The patients infected with COVID-19 pre-T/C were significantly younger than the patients infected with COVID-19 post-T/C (median 54.0 years versus 60.5 years; Mann-Whitney Wilcoxon two-sample test p = 0.008). Similar proportions of the pre-T/C versus post-T/C COVID-19 cases were male (55.2% versus 56.9%). Among the pre-T/C cases, 46/105 (43.8%) had hematologic malignancies or were bone marrow transplant recipients, 43/105 (41.0%) were solid organ transplant recipients, and the remaining 16/105 (15.2%) had other forms of immunosuppression. Among the post-T/C patients, 56/102 (54.9%) had hematologic malignancies or were bone marrow transplant recipients, 35/102 (34.3%) were solid organ transplant recipients, and 11/102 (10.8%) had other forms of immunosuppression. The differences in distribution by gender and underlying condition were not statistically significant.

Of the 102 patients who developed COVID-19 post-T/C, the median elapsed time to COVID-19 positivity was 79 days following T/C administration (range 1 to 204 days). Five persons in this group had a positive test within 9 days of receiving T/C (one on day 1, two on day 3, one on day 7, and one on day 9) thus potentially having received T/C as post-exposure, rather than pre-exposure, prophylaxis unintentionally. Of the remaining 97 cases, half occurred within 11 weeks of receiving T/C, with 75% occurring within 14 weeks and 90% occurring within 17 weeks.

Vaccine status and treatment with therapeutic agents differed significantly between pre-T/C and post-T/C COVID-19 cases. Patients infected pre-T/C were more likely to have not received any doses of COVID-19 vaccine (14.3% versus 2.9%); conversely the post-T/C cases were more likely to have had received at least three vaccine doses (72.2% versus 58.1%). The difference in vaccination status was statistically significant (p < 0.001). Of patients with pre-T/C infections, the majority were treated with a therapeutic agent, although a significantly higher percentage of the post-T/C group received a therapeutic agent compared to the pre-T/C group (80/105 (76.2%) versus 93/102 (91.2%) p = 0.004). In the pre-T/C group, the most commonly used agents were sotrovimab (57/105; 54.3%) and remdesivir (18/105; 17.1%), and 5/105 (4.8%) received nirmatrelvir-ritonavir, while in the post-T/C group, the most common agents were nirmatrelvir-ritonavir (40/102; 39.2%), bebtelovimab (34/102; 33.3%), and remdesivir (17/102; 16.7%).

### Hospitalization

Twenty-six of the 105 (24.8%) patients who developed COVID-19 infection pre-T/C were hospitalized (Table 1), with a median hospital length of stay of 7.5 days (range 1 to 119 days) (Table 2). More than a quarter (26.9%) remained hospitalized for > 30 days. None of the patients died, though seven (26.9%) required an intensive care unit stay. Four cases of hospital-acquired infection occurred among the pre-T/C group and thus COVID-19 was not the primary cause of hospitalization.

**Table 2:** Characteristics of patients hospitalized with COVID-19 infection before and after receipt of T/C.

Patient Characteristic	Hospitalized patients with COVID-19 infection pre-T/C (*n = 26*)	Hospitalized patients with COVID-19 infection post-T/C (*n = 6*)	*p*-value
**Median age [range], years**	53.5 [18–78]	69.2 [59–78]	p = 0.03
**Patient gender**			N.S.
Male (%)	17 (65.4)	4 (66.7)	
Female (%)	9 (34.6)	2 (33.3)	
**Underlying conditions**			N.S.
Solid organ transplantation (%)	15 (57.7)	4 (66.6)	
Hematologic malignancy or bone marrow transplantation (%)	8 (30.8)	1 (16.7)	
Other (%)	3 (11.5)	1 (16.7)	
**Number (%) of vaccine doses received prior to COVID diagnosis** ^**†**^			p<0.0001
0	9 (34.6)	1 (16.7)	
1–2	9 (34.6)	2 (33.3)	
3	7 (26.9)	2 (33.3)	
4	1 (3.8)	1 (16.7)	
**Median Days from most recent vaccine to positive SARS CoV-2 test (range)**	119 (25–310)	224 (30–527)	N.S.
Therapeutics received* **(%)**			p = 0.04
Nirmatrelvir-Ritonavir	0	1 (16.7)	
Bebtelovimab	0	1 (16.7)	
Remdesivir	17* (65.4)	3 (50.0)	
Sotrovimab	5* (19.2)	0 (0.0)	
None	5 (19.2)	1 (16.7)	
**Number of patients with ICU stay (%)**	7 (26.9)	0 (0.0)	N.S.
**Median Hospital length of stay, days** ^**‡**^ **(range)**	7.5 (1–119)	7 (3–16)	N.S.
**Median Days from T/C to positive SARS CoV-2 test**	NA	75 (3–149)	—

^†^One patient’s vaccine history was not documented within the electronic medical record

*One patient was treated with both sotrovimab and remdesivir

^‡^One patient was hospitalized at an outside institution and medical records were unavailable to us

Six (5.9%) of the 102 patients who developed COVD-19 post-T/C were hospitalized, with a median hospital length of stay of 7 days (range 3 to 16 days) (Table 2). None of the five patients for whom duration of stay data were available required hospitalization for more than 30 days. Four of the six hospitalized patients were solid organ transplant recipients, one had hematologic malignancy, and the remaining patient was on immunosuppressive therapy for an underlying autoimmune disease. All six presented with symptoms consistent with a viral respiratory infection, including fever, cough, weakness, and myalgia. Three of the six exhibited hypoxemia, of whom one had a *Legionella* co-infection. No patients in this population required an intensive care unit stay.

The rate of hospitalization among those infected with COVID-19 post-T/C was one-quarter the hospitalization rate of those who developed infection pre-T/C (Table 1) (rate ratio [RR] = 0.24; 95% confidence intervals [CI] = 0.10–0.55; p = 0.0002). The hospitalization rate for post-T/C patients with a hematologic malignancy or bone marrow transplant who developed COVID-19 was one-tenth that of those who had not yet received T/C (RR = 0.10; 95% CI = 0.01–0.72; p = 0.01) (Table 2). For patients who had received solid organ transplants, the corresponding values were RR = 0.33; 95% CI = 0.12–0.90; p = 0.02; for patients with all other conditions, the RR was 0.91, with 95% CI = 0.07–12.1; p = not significant. E-values for the significant associations between underlying condition and hospitalization ranged between 5.51 and 19.49 (S1 Table). The RR for ICU admission could not be calculated, although the difference in ICU admission was significant at the p = 0.008 level.

Those patients hospitalized with infection before receiving T/C were more likely to have been unvaccinated (9/26; 34.6%) compared to those hospitalized after receiving T/C (1/6; 16.7%) (Table 2). Having at least one dose of vaccine strongly protected against hospitalization in both pre- and post-T/C groups (pre-T/C group RR = 0.31, 95% CI = 0.1–0.57; p = 0.02 and post-T/C group RR = 0.15; 95% CI = 0.03–0.94; p = 0.04). The corresponding E-values were 5.91 and 12.81, respectively (S1 Table).

## Discussion

We identified several COVID-19 cases among high-risk patients in our health system who had received T/C for pre-exposure prophylaxis. However, when we compared those who had contracted COVID-19 prior to receiving T/C prophylaxis with those infected after, patients with infection after receiving T/C were only a quarter as likely to be hospitalized for COVID-19 and were also significantly less likely to be admitted to the ICU.

It is difficult to assess, however, the impact of T/C prophylaxis on the lower hospitalization rate in our cohort. Almost all patients who had COVID-19 after receiving prophylactic T/C were also offered additional treatment for symptomatic COVID-19, including updated monoclonal antibodies or antiviral therapies, and nearly three-quarters had received at least three COVID-19 vaccine doses, including 37% who had received four. In addition to the availability of additional vaccine doses and a greater range of effective COVID-19 therapeutics over time, there have also been rapidly changing subvariants of varying severity and increasing immune evasiveness, further complicating the interpretation of therapeutic effectiveness. Monoclonal antibodies targeting the regions of the spike protein of the SARS CoV-2 virus under evolutionary pressure have not been studied at a pace that meets the speed of the evolution of variants in the COVID-19 pandemic. Our study underscores the inherent challenge of assessing real-world efficacy of T/C outside of a clinical trial given the rapidly evolving SARS CoV-2 pandemic.

Our findings differ from those of the initial clinical trial of T/C which had demonstrated an 83% risk reduction for symptomatic COVID-19 infection among recipients of T/C. However, the PROVENT trial largely excluded patients with solid organ transplantation or hematologic malignancy, and less than 4% of the study population was identified as having an immunocompromising condition [3]. Additionally, the trial was conducted prior to the emergence of the Omicron variant, which has become the predominant circulating variant worldwide since December 2021. Indeed, other monoclonal antibodies have been shown to be ineffective against the Omicron variants [4–6, 9]. *In vitro* studies of cilgavimab, one of the two components of T/C, suggested it would maintain some efficacy against the emerging Omicron subvariants, but at a higher concentration than was initially studied in the PROVENT trial. This finding led to the February 2022 FDA recommendation to double the T/C dose.

Since T/C has been in use, several studies have shown T/C recipients to have contracted COVID-19. Among a study of 416 kidney transplant recipients who received T/C in France, there were 39 cases of COVID-19 infection and 2 deaths post-TC, but the drug was dosed at 150 mg of tixagevimab and 150 mg of cilgavimab, supporting that the lower dose was insufficient to provide protection against Omicron [12]. In another study among 222 solid organ transplant recipients, COVID-19 infections were observed after T/C, but it appeared that there were fewer infections and hospitalizations among T/C recipients compared with vaccine-matched controls [13]. This study did not evaluate background community COVID-19 transmission rates, nor use of therapeutics among patients who contracted COVID-19. A larger study of 1112 immunocompromised patients in France noted post T/C Omicron infections including 2 deaths, and noted those with milder disease generally had received additional early therapeutics [14]. A large study from Israel examined the protective role of T/C pre-exposure prophylaxis among 825 immunocompromised patients who received the 150 mg tixagevimab and 150 mg cilgavimab compared to 4299 immunocompromised patients who did not receive drug [15]. Hospitalization, severe disease, and deaths were reduced among T/C recipients. Interpretation of the study is complicated, however, given the different follow-up periods between the two groups (53 days for the T/C recipients vs 73 days for the non-T/C recipients), lack of information regarding therapeutics given, and high death rate within the control group (exceeding hospitalizations).

Our study addresses in detail the therapeutics given in each group and acknowledges how these treatments may have had an important influence on patient outcomes. We also highlight the significant role of the epidemiologic curve in assessing case rates during the Omicron era, and the importance of understanding community transmission rates over time in terms of assessing effectiveness of various interventions.

Our study has several limitations. First, although we used multiple data sources to identify cases, patients receiving COVID-19 care at other health care systems may not have been captured in our data. Second, because the limitations imposed by the use of retrospective data, it was not possible to conduct more rigorous analyses that might have allowed us to determine the relative contributions of T/C, underlying patient conditions, prior immunity to COVID-19, vaccine, circulating variants, and antiviral and monoclonal antibody treatment to the risk of developing COVID-19. However, the associations for which we were able to identify a significant effect in the main analysis had large E-values ranging from 5.51 and 19.49, indicating that the level of association between factors such as prior infection with COVID-19 and both the T/C exposure and outcomes of interests would not plausibly reach such levels of association.

Despite these limitations, our findings demonstrate that high-risk patients who have received T/C pre-exposure prophylaxis continue to develop symptomatic COVID-19 infections in the Omicron era. We note that those were infected with COVID-19 after having received T/C in our study had fewer hospitalizations and ICU stays, they also had more doses of COVID vaccines and received therapeutics predicted to be effective for Omicron. While no patients in our cohort experienced direct harm from receiving T/C, our results raise questions about the ongoing value of T/C pre-exposure prophylaxis. Given the many rapidly changing aspects of the COVID-19 pandemic, including the evolution of variants and subvariants, the protection offered by additional vaccine doses, and the development of effective antivirals and therapeutic monoclonal antibodies, we are unable to truly assess the contribution of T/C to the improved outcomes among patients without a rigorous clinic trial. Such a trial, however, would be difficult to perform and would run the risk of being rapidly outdated. As a result, decisions regarding the significant resources required to administer this drug unfortunately continue to depend on data from *in vitro* studies and expert recommendations. Given the prophylactic nature of the drug, there may have been less scrutiny as to its utilization. However, health systems and recipients of the drug must understand its limitations, especially in the face of changing variants.

## Supporting information

S1 Table Sensitivity analyses based on E-values. (DOCX)

## References

[1] Lauring AS, Tenforde MW, Chappell JD, et al. Clinical severity of, and effectiveness of mRNA vaccines against, covid-19 from omicron, delta, and alpha SARS-CoV-2 variants in the United States: prospective observational study. *BMJ*. 03 09 2022;376:e069761. doi: 10.1136/bmj-2021-069761 35264324 PMC8905308

[2] Malahe SRK, Hoek RAS, Dalm VASH, et al. Clinical characteristics and outcome of immunocompromised patients with COVID-19 caused by the Omicron variant: a prospective observational study. *Clin Infect Dis*. Jul 23 2022; 10.1093/cid/ciac571 PMC9384537 35869843

[3] Levin MJ, Ustianowski A, De Wit S, et al. Intramuscular AZD7442 (Tixagevimab-Cilgavimab) for Prevention of Covid-19. *N Engl J Med*. 06 09 2022;386(23):2188–2200. doi: 10.1056/NEJMoa2116620 35443106 PMC9069994

[4] Cao Y, Wang J, Jian F, et al. Omicron escapes the majority of existing SARS-CoV-2 neutralizing antibodies. *Nature* Feb 2022;602(2898):657–663. doi: 10.1038/s41586-021-04385-3 35016194 PMC8866119

[5] VanBlargan LA, Errico JM, Halfmann PJ, et al. An infectious SARS-CoV-2 B.1.1.529 Omicron virus escapes neutralization by therapeutic monoclonal antibodies. *Nat Med*. 03 2022;28(3):490–495. doi: 10.1038/s41591-021-01678-y 35046573 PMC8767531

[6] Dejnirattisai W, Huo J, Zhou D, et al. SARS-CoV-2 Omicron-B.1.1.529 leads to widespread escape from neutralizing antibody responses. *Cell*. 02 03 2022;185(3):467–484.e15. doi: 10.1016/j.cell.2021.12.046 35081335 PMC8723827

[7] AstraZeneca Media Statement. https://www.astrazeneca.com/media-centre/statements/2022/fda-evusheld-dosage-update-us.html Accessed December 13, 2022.

[8] National Institutes of Health. COVID-19 Treatment Guidelines Panel. Coronavirus Disease 2019 (COVID-19) Treatment Guidelines. https://www.covid19treatmentguidelines.nih.gov/ Accessed August 25, 2022. 34003615

[9] Fiaschi L, Dragoni F, Schiaroli E, et al. Efficacy of Licensed Monoclonal Antibodies and Antiviral Agents against the SARS-CoV-2 Omicron Sublineages BA.1 and BA.2. *Viruses*. 06 23 2022;14(7) doi: 10.3390/v14071374 35891355 PMC9321742

[10] Mathur M. B., Ding P., Riddell C. A., & VanderWeele T. J. (2018). Website and R package for computing E-values. Epidemiology (Cambridge, Mass.), 29(5), e45. doi: 10.1097/EDE.0000000000000864 29912013 PMC6066405

[11] VanderWeele T. J., & Ding P. (2017). Sensitivity analysis in observational research: introducing the E-value. Annals of internal medicine, 167(4), 268–274 doi: 10.7326/M16-2607 28693043

[12] Benotmane I, Velay A, Gautier-Vargas G, et al. Breakthrough COVID-19 cases despite prophylaxis with 150 mg of tixagevimab and 150 mg of cilgavimab in kidney transplant recipients. *Am J Transplant*. Jun 17 2022; doi: 10.1111/ajt.17121 35713984 PMC9350296

[13] Al Jurdi A, Morena L, Cote M, Bethea E, Azzi J, Riella LV. Tixagevimab/cilgavimab pre-exposure prophylaxis is associated with lower breakthrough infection risk in vaccinated solid organ transplant recipients during the omicron wave. *Am J Transplant*. Jun 21 2022; doi: 10.1111/ajt.17128 35727916 PMC9906353

[14] Nguyen Y, Flahault A, Chavarot N, et al. Pre-exposure prophylaxis with tixagevimab and cilgavimab (Evusheld©) for COVID-19 among 1112 severely immunocompromised patients. *Clin Microbiol Infect*. Aug 01 2022 doi: 10.1016/j.cmi.2022.07.015 35926762 PMC9340091

[15] Kertes S, David SSB, Engel-Zohar N, et al. Association between AZD7442 (Tixagevimab-Cilgavimab) administration and severe acute respiratory syndrome coronavirus 2 (SARS-CoV-2) infection, hospitalization, and mortality. *Clin Inf Dis*. July 29 2022;ciac625. 10.1093/cid/ciac625 PMC9384583 35904210

