# Supplementary material for: Real world effectiveness of tixagevimab/cilgavimab (Evusheld) in the Omicron era
Source: PLoS One. 2023 Apr 27;18(4):e0275356. doi: 10.1371/journal.pone.0275356 (PMC10138227; doi:10.1371/journal.pone.0275356)
Supplement: S1 Table — (DOCX) [file pone.0275356.s001.docx]

Supplemental table. **Sensitivity analyses based on E-Values**

| Outcome | Rate Ratio (RR) and 95% CI | E-Value* |
| --- | --- | --- |
| Hospitalization for patients receiving T/C pre-exposure prophylaxis | 0.24 [0.10-0.55] | 7.8 |
| Hospitalization for patients with hematologic malignancy | 0.10 [0.01-0.72] | 19.49 |
| Hospitalization for patients that received solid organ transplants | 0.33 [0.12-0.90] | 5.51 |
| Hospitalization for patients with all other conditions | 0.91 [0.07-12.1] | 1.43 |
| Hospitalization for pre-T/C patients with at least one dose of vaccine | 0.31 [0.10-0.57] | 5.91 |
| Hospitalization for post-T/C patients with at least one dose of vaccine | 0.15 [0.03-0.94] | 12.81 |

* The E-Value computes the amplitude of an unmeasured confounder to explain away the observed association
